# Oblique lateral interbody fusion combined with unilateral versus bilateral posterior fixation in patients with osteoporosis

**DOI:** 10.1186/s13018-023-04262-x

**Published:** 2023-10-16

**Authors:** Xiang Ma, Longwei Lin, Jian Wang, Lin Meng, Xingze Zhang, Jun Miao

**Affiliations:** 1 Department of Spine Surgery, Tianjin Hospital, Tianjin University, Jiefangnanlu 406, Hexi District, Tianjin, 300210 China; 2 Tianjin Medical University, Tianjin, China; 3 Tianjin University, Tianjin, China

**Keywords:** Pedicle screw internal fixation, Osteoporosis, Oblique lumbar interbody fusion, Fusion rate, Cage subsidence

## Abstract

**Purpose:**

To compare the clinical efficacy of oblique lateral interbody fusion (OLIF) combined with unilateral (UPSF) and bilateral pedicle screw internal fixation (BPSF) in patients with osteoporosis.

**Methods:**

Clinical data of 57 patients who underwent single-segment OLIF surgery with a clear diagnosis of osteoporosis from December 2018 to May 2021 were retrospectively analyzed, of which 27 patients underwent OLIF + UPSF and 30 patients underwent OLIF + BPSF. Surgical technique-related indexes were recorded, including operative time, operative blood loss and postoperative hospital stay; clinical outcome-related indexes included postoperative complications, Visual analogue scale (VAS) and Oswestry disability index (ODI) at preoperative, 1 week, 1 month, 3 months, and 12 months postoperative follow-up; and imaging outcome-related indexes included the measurement of preoperative and postoperative segmental lordosis (SL), and observation of the degree of cage subsidence and bone graft fusion.

**Results:**

The surgery was successfully performed in 57 patients, and there was no statistical difference in operative blood loss and postoperative hospital stay between UPSF group and BPSF group (*P* > 0.05). In terms of operative time, there was a significant difference (UPSF group: 92.30 ± 11.03 min, BPSF group: 119.67 ± 16.41, *P* < 0.05). Postoperative VAS and ODI scores exhibited significant improvement (*P* < 0.05). At the 12 months postoperative follow-up, the VAS and ODI scores in the BPSF group were significantly better than those in the UPS group (*P* < 0.05). Compared with the preoperative images, the SL was significantly improved in both groups after surgery (*P* < 0.05). At 6 months postoperatively, the fusion rate in the UPSF group was significantly lower than that in the BPSF group (*P* < 0.05). At 1 year postoperatively, the fusion rate in the UPSF group was not significantly different from that in the BPSF group (*P* > 0.05). At 1 year postoperatively, the rate and degree of cage subsidence was higher in the UPSF group than in the BPSF group (*P* < 0.05).

**Conclusion:**

In the long term, OLIF combined with bilateral posterior fixation applied to the osteoporosis patients is superior to OLIF surgery combined with unilateral posterior fixation in terms of clinical and imaging outcomes. It is effective in improving pain relief and functional improvement, accelerating bone graft fusion, and reducing cage subsidence compared with UPSF.

## Introduction

In recent years, osteoporosis has become a global health concern. It is primarily prevalent among individuals in their middle to advanced years and manifests as low bone density, impairment of the microstructure of bone tissue, and limited bone regeneration [1, 2].At the same time, Lumbar degenerative disease (LDD) stands as a prevalent source of lumbar pain and a surgical intervention for individuals who have gracefully entered the age of 65 and beyond [3]. The frequent co-occurrence of osteoporosis and LDD in the elderly has led to the increasing importance of osteoporosis in orthopedic surgery.

With the rapid development of minimally invasive spinal techniques, OLIF has become one of the main modalities for LDD due to the advantages of low trauma, good patient tolerance, fast pain relief, and effective deformity correction. This procedure exposes the lumbar spine through a lateral peritoneal approach, avoiding the destruction of the posterior vertebral structures in the traditional posterior approach. It allows the placement of larger fusion devices between the vertebral bodies to restore the height of the intervertebral space, increase tension in the posterior longitudinal ligament, expand the volume of the spinal canal, and increase the area of the lateral recess, thereby achieving indirect decompression and relieving neurological symptoms. Macki et al. [4] conducted a systematic review and meta-analysis of 21 studies on cage subsidence after OLIF surgery. The results showed that out of 1,362 cases, 141 cases experienced implant subsidence, with an overall incidence rate of 10.3%. Implant subsidence was identified as the most common complication following oblique lumbar interbody fusion.

In patients with osteoporosis, their reduced bone density results in a lower capacity to withstand maximum stress. To prevent excessive occurrence of implant subsidence, it is widely accepted that additional internal fixation should be applied during OLIF surgery for individuals with osteoporosis. Currently, the most effective and commonly used internal fixation method in lumbar spine surgery is posterior pedicle screw fixation, due to its superior biomechanical stability and ability to effectively promote fusion [5].

Even though there have been numerous studies conducted on the fixation of unilateral versus bilateral posterior approaches, as far as we know, the results of these studies are still controversial and primarily applicable to patients with normal bone density [6–11]. Literature has reported that the biomechanical performance of bilateral pedicle screw fixation is superior to that of lateral fixation and unilateral pedicle screw fixation in OLIF surgery [8]. Hiyama et al. found severe cage subsidence was more common in the UPS group, which suggests that BPS fixation after LLIF may be a better choice over the long term [6]. However, a biomechanical study on 1–2 level lumbar degenerative diseases, comparing OLIF + UPSF and OLIF + BPSF, showed comparable biomechanical stability between the two approaches [11]. Wen et al. found in a retrospective study that OLIF + UPSF is an effective and reliable option for single-level lumbar diseases [7]. Furthermore, some studies have even found a lower incidence rate of adjacent segment disease in the UPSF group, highlighting its advantages [9, 10].

Therefore, the optimal approach for pedicle screw fixation in the population with osteoporosis when applying OLIF remains unclear. In this study, a retrospective comparative study was conducted to investigate the clinical efficacy between UPSF and BPSF in the osteoporosis population, aiming to provide evidence for the clinical application of OLIF combined with PSF technique.

## Materials and methods

**Fig. 1:**
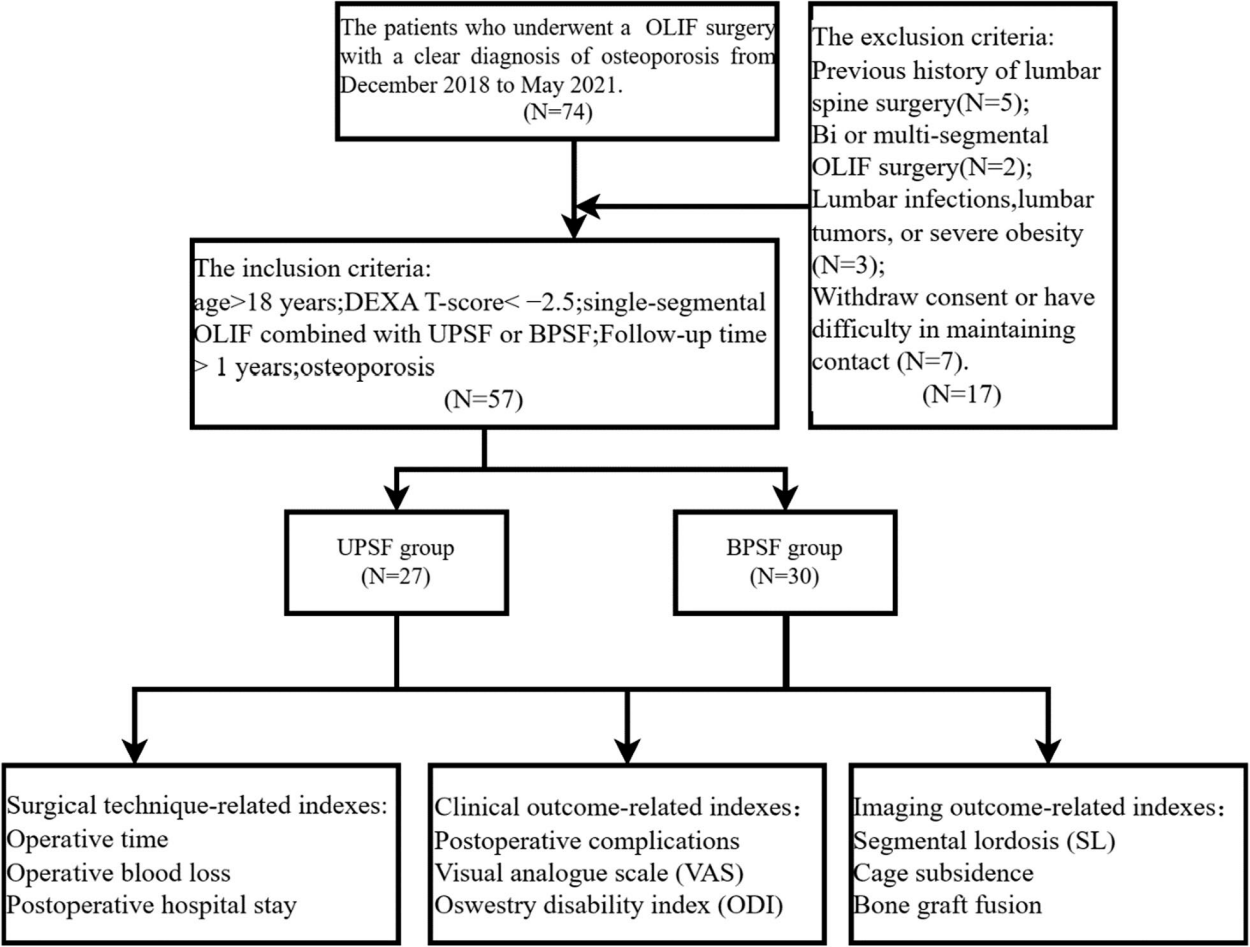
Flow chart of the study design

The ethical approval of this retrospective comparative trial was obtained from the Ethics Committee of Tianjin Hospital, Tianjin University (No. 2023YLS136), and verbal and written informed consent was obtained from all the participants before taking part. This study was reported according to the STROCSS criteria [12]. The study design and subjects are presented in Fig. 1.

### Inclusion and exclusion criteria

The medical records of inpatients who underwent OLIF in our department from 2018 to 2021 were retrospectively analyzed.

The inclusion criteria: (1) Age > 18 years at the time of surgery;(2) The T-score derived from dual-energy X-ray absorptiometry (DEXA) was used to assess bone mineral density (BMD) in the lumbar spine, specifically the L2-L4 region. T-score <  − 2.5;(3) The surgical method was single-level OLIF combined with UPSF or BPSF; (4) Follow-up time > 1 years.

The exclusion criteria:(1) Previous history of lumbar spine surgery; (2) Bi or multi-segmental OLIF surgery;(3) Lumbar infections, lumbar tumors, or severe obesity(BMI > 35 kg/m^2^);(4) Patients withdraw consent or have difficulty in maintaining contact during follow-up.

**Fig. 2:**
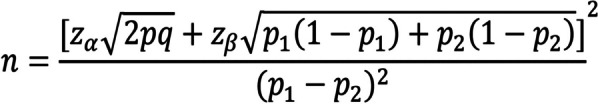
The formula used for calculating the sample size in this retrospective comparative study

This study is a retrospective comparative study, with the cage subsidence serving as the primary outcome measure. Based on the findings from the previous study, p1(the incidence rate of UPSF) is 0.375, while p2 (the incidence rate of BPSF) is 0.095 [6]. With a 5% significance level and 80% power, the sample size ratio between two groups is 1:1. Referring to the formula (Fig. 2), the calculated sample size for each group is 29 cases.

### Surgical procedures

The criteria for patient selection for unilateral or bilateral fixation were established based on certain factors. These factors included the patient’s desires, patient’s age, overall health condition, comorbidities, and the surgeon’s expertise and preference. All patients are presented with a detailed explanation of the surgical procedure by experienced spinal surgeons, and their consent is obtained.

The surgical details of OLIF were performed based on standard procedure [13, 14]. After general anesthesia, the patient was placed in the right lateral recumbent position, and the target vertebral space was located under C-arm X-ray. The external oblique, internal oblique, and transversus abdominis muscles were separated bluntly along the muscle fibers. The transversus abdominis fascia was bluntly dissected into the retroperitoneal space, and the extraperitoneal fat was pushed ventrally. The anterior border of the psoas major muscle was pulled or peeled posteriorly to reveal the left anterior lumbar disc in the space between the psoas major muscle and the abdominal aorta, and a positioning needle was placed. The working channel was installed and properly propped to fully expose the target space. The disc was dissected, and the nucleus pulposus and upper and lower cartilage endplates were removed with a reamer and spatula, and the contralateral annulus fibrosus was released. The intervertebral space was opened to the appropriate level using trial molds, and a suitable anterior lumbar fusion device filled with autologous bone or allogeneic bone was selected for placement in the intervertebral space. After X-ray fluoroscopy confirmed that the fusion device was satisfactorily positioned, the surgical wound was meticulously sutured layer by layer and a drainage tube was placed or not depending on the intraoperative bleeding. The patient's position was changed to prone, and UPSF or BPSF was selected according to the signed informed consent form.

### Outcome indexes

All patients in the trial were examined preoperatively, including radiography, magnetic resonance imaging (MRI) and computed tomography (CT), to clarify diagnosis. They were also investigated at 1 week, 1 month, 3 months, 12 months postoperatively, and completed the appropriate imaging examinations and questionnaires.

Surgical technology-related data: Operative time, operative blood loss and postoperative hospital stay were essential factors that were recorded to assess the overall condition of the surgery.

Clinical outcomes: The patients' leg/back pain symptoms and functional improvement were assessed by Visual analogue scale (VAS) and Oswestry disability index (ODI) at preoperative, 1 week, 1 month, 3 months, and 12 months postoperative follow-up, respectively [6, 13, 15]. Complications were recorded during the follow-up [6, 13, 15].

**Fig. 3:**
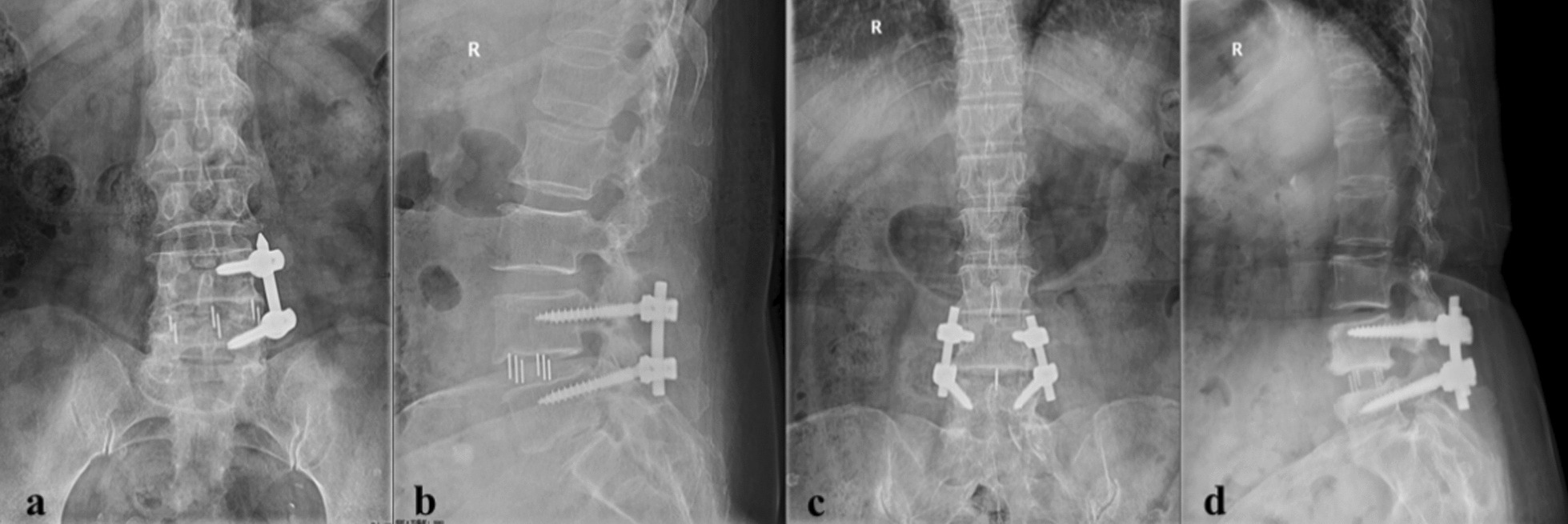
Anteroposterior and lateral radiographs at 1 month postoperatively in each group: **a**, **b** UPSF group **c**, **d** BPSF group

Imaging outcomes: Anteroposterior and lateral of the lumbar spine were performed at preoperative and postoperative follow-up (Fig. 3). Two spinal surgeons individually measured radiographic data using Surgimap software and calculated the average of their results. (1) Segmental lordosis (SL) was defined as the angle formed by the lower endplate of the upper vertebrae and the upper endplate of the lower vertebrae. (2) According to Marchi et al.’s study [16], cage subsidence was categorized into grades 0-III according to intervertebral space height (ISH) immediately after surgery: grades 0-III corresponded to ISH decreases of 0–24%; 25–49%; 50–74%; and 75–100%, respectively. ISH was the average of the anterior and posterior ISH. (3) The interbody fusion grading system, as proposed by Bridwell et al.[17], enabled a comprehensive evaluation of fusion outcomes. It was segregated into 4 levels. Grade I, fusion with remodeling and trabeculae present; Grade II, graft intact, incomplete remodeling and fusion, but no translucency; Grade III, graft intact, with potential translucency at the top and bottom of the graft; Grade IV, fusion absent, graft collapsed/resorbed.

### Statistical analysis

Statistical analysis and graph production were conducted utilizing GraphPad Prism (version 8.0 for Windows, GraphPad Software, San Diego, California USA). Numerical data are expressed as mean ± standard deviation (Mean ± SD), and the comparisons between two groups and the differences within each group before and after the surgery will be assessed using independent two-sample *t*-test and paired t-test, respectively. Qualitative data are presented in the form of number (%). The differences between groups were compared using the chi-square or Fisher exact test. Wilcoxon Mann–Whitney test was used to analyze ranked data. *P* < 0.05 was considered statistically significant.

## Results

### Baseline data

The baseline data has been summarized in Table 1. From December 2018 to May 2021, our study encompassed a collective of 57 patients who were carefully selected according to specific inclusion and exclusion criteria. Among them, 27 patients received OLIF + UPSF treatment, while 30 patients received OLIF + BPSF treatment. No significant differences existed between the two groups concerning age, gender, BMI, American Society of Anesthesiologists (ASA), operative site, and follow-up time (*p* > 0.05, Table 1).

**Table 1:** Baseline data of included patients

Characteristics	UPSF group	BPSF group	X2/t	p
Age (year), mean ± SD	63.29 ± 6.97	64.10 ± 6.69	0.444	0.659
Gender (n, %)	27	30	0.198	0.656
Male	5(18.5%)	7(23.3%)		
Female	22(81.5%)	23(76.7%)		
BMI (kg/m2), mean ± SD	25.93 ± 2.97	25.87 ± 2.70	0.073	0.942
ASA (n, %)			1.721	0.613
Grade 1	26(96.3%)	26(86.7%)		
Grade 2	1(3.7%)	3(10%)		
Grade 3	0	1(1.8%)		
Operative site(n, %)			1.417	0.234
L3/4	21(77.8%)	19(63.3%)		
L4/5	6(22.2%)	11(36.7%)		
Follow-up time (months)	29.15 ± 3.91	27.87 ± 3.00	1.396	0.168

### Surgical technology-related data

Surgical technology-related data has been summarized in Table 2. There was no significant difference (*P* > 0.05) between the UPSF and BPSF groups in terms of operative blood loss and postoperative hospital stay. However, the BPSF group had a longer average operation time compared to the UPSF group (UPSF:92.30 ± 11.03; BPSF:119.67 ± 16.41; *p* < 0.05; Table 2).

**Table 2:** Surgical technology-related data

Characteristics	UPSF group	BPSF group	t	p
Operative time(min)	92.30 ± 11.03	119.67 ± 16.41	7.454	< 0.001
operative blood loss(ml)	97.04 ± 37.29	114.00 ± 35.58	1.757	0.085
postoperative hospital stay (d)	6.15 ± 3.08	6.20 ± 1.82	0.076	0.94

### Clinical outcomes

**Fig. 4:**
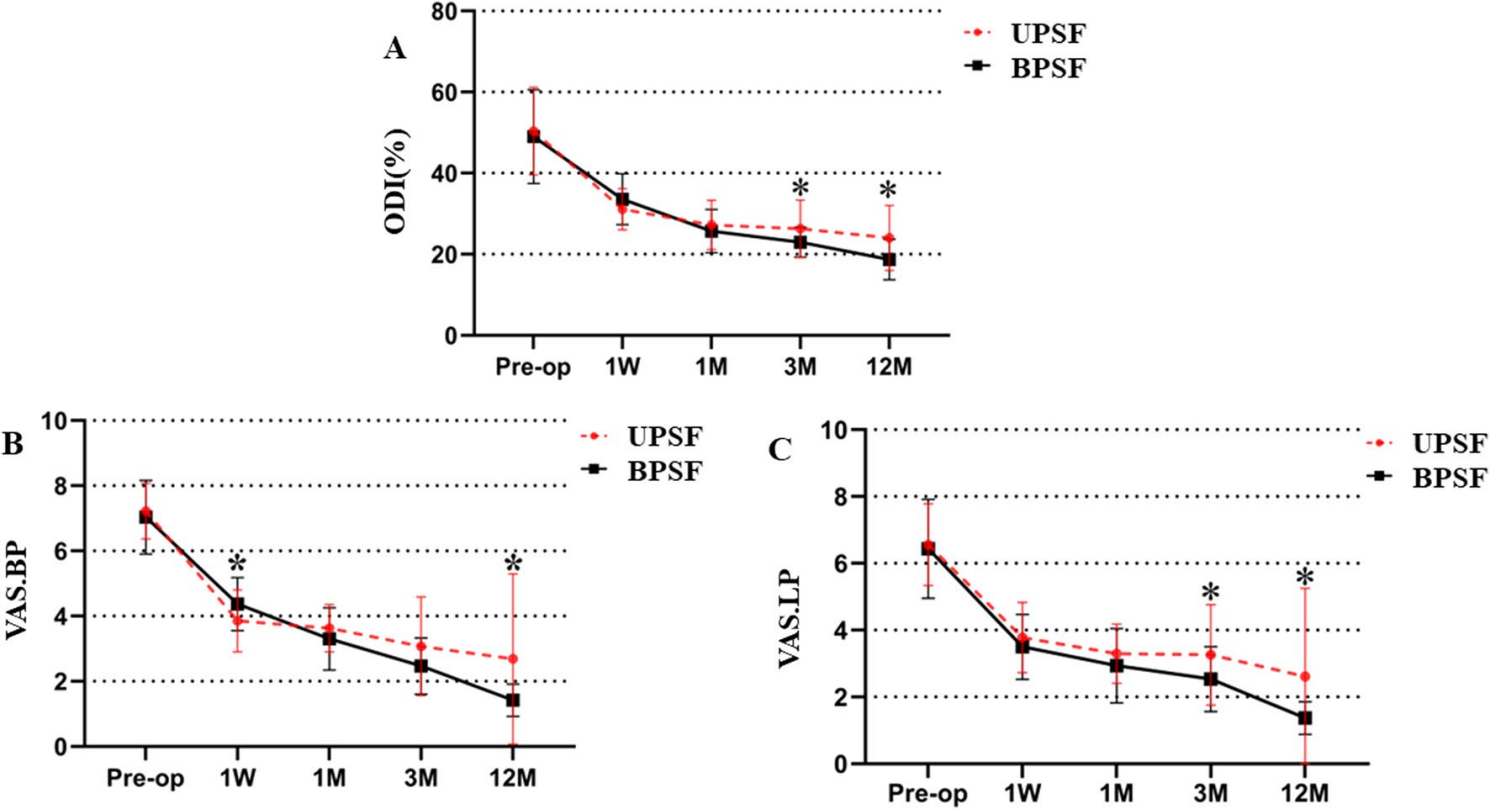
Comparison of ODI and VAS scores between UPSF group and BPSF group from preoperative to postoperative 12 months. **A**, ODI (%) ranging from 0 (non-disabled) to 100 (highly disabled). **B**, VAS.BP (VAS for back pain score), ranging from 0 (no pain) to 10 (most severe pain).**C**, VAS.LP(VAS for leg pain score), ranging from 0 (no pain) to 10 (most severe pain). *P < 0.05

There was no significant difference observed in the preoperative ODI and VAS scores between two groups (*p* > 0.05, Fig. 4). During the postoperative period, both the VAS and ODI scores of both groups were significantly lower compared to preoperative scores (*p* < 0.05). At 1 week postoperatively, the UPSF group exhibited significantly lower scores in VAS for lower back pain compared to the BPSF group (*p* = 0.033). At the 3 months postoperatively, the BPSF group had significantly lower scores in both ODI and VAS for leg pain compared to the UPSF group (*p* = 0.038, *p* = 0.034 respectively). Finally, at the 12 months postoperatively, the BPSF group had significantly lower scores in both ODI and VAS compared to the UPSF group (all *p* < 0.05).

### Imaging outcomes

The imaging outcomes of the two groups has been summarized in Table 3. There was no significant difference in preoperative SL angle between the two groups. The postoperative SL angle has been significantly improved compared to preoperative data (*P* < 0.05). At 1 year postoperatively, 5 patients in the UPSF group showed Grade I subsidence, while 1 patient in the UPSF group had Grade I subsidence. Additionally, one patient in the UPSF group had Grade II subsidence. There was a significant difference in the overall distribution of cage subsidence between the two groups (z = 2.162, P = 0.031). According to the Bridwell’s fusion grading system, bone graft fusion is classified into four grades [17]. Grades I and II indicate successful fusion, while grades III and IV represent fusion failure [13, 17]. At 6 months postoperatively, the fusion rate in the UPSF group was 10/27, compared to 22/30 in the BPSF group. There was a statistically significant difference in the fusion rates among different grades between the two groups (X^2^ = 7.8, *P* = 0.034). At 1 year postoperatively, the fusion rate in the UPSF group was 23/27, while in the BPSF group it was 29/30. No significant difference in the fusion rates among different grades was observed between the two groups (X^2^ = 3.663, *P* = 0.275).

**Table 3:** Imaging outcomes.

Characteristics	UPS	BPS	X2/t/W M-W	p
Preoperative SL°	4.33 ± 1.25	4.86 ± 2.41	1.052	0.298
Postoperative SL°	7.18 ± 1.05*	7.06 ± 1.01*	0.350	0.728
Fusion rate at 6 months(n, %)			7.8	0.034
Grade I	3(11.1%)	7(23.3%)		
Grade I	7(25.9%)	15(50.0%)		
Grade III	16(59.3%)	8(26.7%)		
Grade IV	1(3.7%)	0		
Fusion at 12 months(n, %)			3.663	0.275
Grade I	18(66.7%)	26(86.7%)		
Grade II	5(18.5%)	3(10.0%)		
Grade III	3(11.1%)	1(3.3%)		
Grade IV	1(3.7%)	0		
Cage subsidence at 1 year postoperatively			2.162▲	0.031
Grade 0	21	29		
Grade I	5	1		
Grade II	1	0		
Grade III	0	0		

*represents a statistically significant difference (p < 0.05) compared to preoperative measurements; ▲,comparison of cage subsidence at 1 year postoperatively was conducted by Wilcoxon Mann–Whitney test

## Discussion

OLIF is a globally popular minimally invasive technique for lumbar spine surgery. It was first introduced by Mayer in 1997 [18], and officially named and reported by Silvestre in 2012 [19]. The OLIF procedure utilizes the anatomical gap between the aorta/inferior vena cava (IVC) and the psoas muscle to access the intervertebral disc space, without the need to open the vertebral canal, thus preserving the integrity of the posterior muscles, ligaments, and bony structures. Additionally, this technique allows for the removal of a substantial amount of intervertebral disc tissue, resulting in increased surface contact between the fusion device and the endplate. Consequently, the OLIF technique and its principles have rapidly gained international recognition and become an advanced and innovative minimally invasive surgical approach in the field of spine surgery, particularly for the treatment of lumbar spine disorders.

According to a retrospective study by Tempel, a significant predictor of subsidence in fusion surgery is decreased bone density [20]. The reduction in bone density increases the risk of endplate damage during OLIF surgery, leading to iatrogenic subsidence. For patients with a bone density T-score less than -1.0, posterior percutaneous pedicle screw fixation can help prevent cage subsidence [20]. It is undisputed that additional external fixation should be applied during OLIF surgery for individuals with osteoporosis. However, the decision regarding the use of UPS or BPS fixation in lumbar fusion surgery remains controversial. In OLIF surgery, BPS is considered the traditional standard fixation method due to its excellent biomechanical stability. Nonetheless, the inflexibility of BPS fixation could potentially contribute to a greater prevalence of instrument-associated osteoporosis [21]. Additionally, BPS fixation can lead to adjacent segment degeneration due to the additional pressure exerted on neighboring segments [9, 10]. Moreover, in contrast to UPS fixation, BPS fixation entails drawbacks such as increased invasiveness, escalated medical expenses, and heightened impairment to posterior structures. [22–24].

In this study, we initially compared the surgical technology-related data brought by two different degrees of fixation. The experimental findings reveal that only the mean operation time presented significant difference. This can be attributed to the fact that bilateral fixation entails more surgical procedures. Several other retrospective studies have also shown that unilateral fixation can achieve shorter surgical durations [7, 23]. However, there were no apparent differences in operative blood loss and postoperative hospital stay. This discovery is inconsistent with preceding reports [23, 24]. One possible reason for this disparity is the utilization of percutaneous pedicle screw fixation in OLIF [7]. In comparison to previous surgical techniques, such as PLIF, this insertion technique is relatively minimally invasive.

In our investigation, we have discovered that UPSF, in comparison to BPSF, offers certain advantages in the early post-operative period, particularly evident in VAS for lower back pain. This could be attributed to the minimal damage UPS inflicts on the paraspinal muscles [13, 25], resulting in superior relief of lumbar pain and improved lumbar spine function in the UPSF group. However, this advantage is not sustained beyond 1 month. After one month, the paravertebral muscles of all patients had entered the stage of recovery, with no significant differences observed between the two groups [13]. Furthermore, due to the increased rehabilitation activities of the patient, it appears that UPSF is inadequate. The experimental findings also indicate that patients with osteoporosis require an extended period of rehabilitation after UPSF. It is recommended to appropriately delay physical activities and decrease their intensity. According to the guidelines for postoperative rehabilitation, wearing rigid orthoses for a minimum of three months is recommended [26, 27]. However, based on the clinical outcomes data obtained from patients three months post-operation, it is evident that UPSF group experience noticeably worse leg/back pain symptoms and functional improvement compared to BPSF group, with four individuals experiencing thigh numbness and one patient exhibiting lumbar muscle weakness. The outcome differs from previous retrospective studies, potentially attributed to the inclusion of participants specifically selected for their osteoporotic characteristics in this particular study [28, 29].

Currently, it is widely acknowledged by scholars that a lower bone mineral density increases the risk of sinkage of the fusion device, especially for patients with severe osteoporosis [20, 30]. This is primarily due to insufficient vertebral strength, resulting in endplate collapse and an increased probability of fusion device sinkage [31]. Loss of intervertebral height and fusion device sinkage are the main causes of failure in lateral surgery. When the fusion device sinks, the corresponding vertebral segment experiences significant loss in the area of lateral recess, resulting in a narrowing of the sagittal diameter of the spinal canal and reduced tension in the posterior longitudinal ligament, which may lead to neurological symptoms in the lower limbs. Posterior percutaneous pedicle screw fixation is currently an effective additional fixation method in OLIF surgery, which can effectively maintain three-column stability and reduce surgical complications [13, 14]. Cage subsidence has been observed in both UPSF and BPSF groups, but the degree of sinkage is significantly smaller in the BPS group. A prospective randomized study obtained similar results in a short-term follow-up of 33 patients [6]. These results partially explain the differences between the UPSF and BPSF groups in terms of postoperative symptoms and functional improvement.

Previous study has reported that lower Hounsfield units (HU) on preoperative CT are associated with cage subsidence after LLIF [32–34]. Thus, for patients with low bone density, surgeons should predict the risk of cage subsidence through preoperative HU measurements. Furthermore, they should implement proactive treatment for osteoporosis and consider utilizing BPSF in OLIF surgery, in order to avoid excessive endplate damage and/or disc subsidence [32–34].

Interbody fusion necessitates a stable local environment [29]. The effects of using UPSF and BPSF on maintaining biomechanical stability are inconsistent. UPSF may not provide the same level of stability as BPSF in resisting axial rotation and lateral bending [35]. Compared to BPSF, UPSF involves unilateral internal fixation, which may result in centrifugal rotation [36]. Theoretically, biomechanical stability potentially impacts fusion rates, thus promoting fusion. In this study, we observed differences between the two groups in relation to fusion speed.

In the healthy population, the lumbar lordosis of the L4-S1 segment accounts for two-thirds of the total lumbar lordosis, hence requiring a significant restoration of SL during lower lumbar surgeries [37]. And in short-segment lumbar fusion surgeries, the restoration of appropriate lumbar lordosis remains relatively important [38, 39]. When placing a sufficiently high cage within the intervertebral disc space, choosing the anterior one-third of the position can optimize the restoration of SL and provide indirect neural decompression [40, 41]. Previous study has reported posterior fixation can provide an additional correction of approximately 1° of lumbar lordosis compared to the stand-alone OLIF technique [42]. This may be attributed to the compressive action of the pedicle screw system in posterior fixation, which allows for re-aggregation of the separated facet joints, thereby facilitating the restoration of sagittal lordosis. Our study demonstrates that the combination of OLIF with unilateral or bilateral posterior fixation surgeries both improve the force line of the lumbar segment, with no significant difference between the two approaches in this study. Therefore, both unilateral and bilateral posterior fixation combined with OLIF procedures can achieve satisfactory sagittal lordosis correction capabilities in osteoporotic patients.

There are certain limitations to our research that should be acknowledged. Firstly, this study is a single-center retrospective study, which inherently carries limitations in terms of sample size and may impact the overall credibility of the findings. Secondly, the follow-up duration was relatively short, and longer-term follow-up is necessary to further validate the results. Thirdly, the choice of surgical approach was determined by the collaboration between the spinal surgeon and the patient based on individual needs. Due to the lack of complete randomization in the grouping process, the experimental outcomes were somewhat influenced. Lastly, variations in surgical techniques among different spine surgeons may introduce disparate treatment outcomes.

## Conclusion

For patients with osteoporosis, whether OLIF combined with UPSF or BPSF is utilized, satisfactory clinical outcomes can be achieved in pain relief and functional improvement, as well as restoration of lumbar lordosis. UPSF offers shorter surgical duration and better short-term patient experience postoperatively. However, in the long run, BPSF can lead to faster bone fusion and lower likelihood of cage subsidence. Therefore, bilateral pedicle screw fixation serves as an effective and reliable additional posterior fixation option for osteoporotic patients undergoing OLIF surgery.

## Data Availability

The datasets generated and analyzed during the current study are available from the corresponding author on reasonable request.

## Abbreviations

OLIF: Oblique lateral interbody fusion
UPSF: Unilateral pedicle screw internal fixation
BPSF: Bilateral pedicle screw internal fixation
ODI: Oswestry disability index
VAS: Visual analogue scale
SL: Segmental lordosis
LDD: Lumbar degenerative disease
MRI: Magnetic resonance imaging
CT: Computed tomography
ASA: American Society of Anesthesiologists
IVC: Inferior vena cava
HU: Hounsfield units
